# Prevalence and risk factors of cardiometabolic disease among informal sector workers in Cape Coast, Ghana: Insights from the CarMeR study

**DOI:** 10.1371/journal.pone.0359530

**Published:** 2026-09-29

**Authors:** Thomas Hormenu, Ebenezer Oduro Antiri, Eric Awlime-Ableh, Juliet Elikem Paku, Edward Edem Nartey, Shaibu Bukari, Adeladza Kofi Amegah

**Affiliations:** 1 Department of Health, Physical Education and Recreation, University of Cape Coast, Cape Coast, Ghana; 2 Cardiometabolic Epidemiology Research Laboratory, University of Cape Coast, Cape Coast, Ghana; 3 Department of Labour and Human Resource Studies, University of Cape Coast, Ghana; 4 Department of Biomedical Sciences, University of Cape Coast, Cape Coast, Ghana; Tecnologico de Monterrey, MEXICO

## Abstract

**Background:**

The prevalence of cardiometabolic diseases like obesity, hypertension, diabetes, and dyslipidaemia is increasing rapidly, particularly in low- and middle-income countries (LMICs). Informal workers in Ghana, who account for the majority of the national workforce, contribute significantly to the country’s socioeconomic growth. Often, their work predisposes them to cardiometabolic diseases. This study examines the cardiometabolic burden, its associated factors, and the diagnostic heterogeneity among this unique population.

**Methods:**

This study used cross-sectional data on 948 informal workers (traders, fisherfolk, artisans, farmers, and drivers) in the Cape Coast Metropolis, Ghana. Sociodemographic, lifestyle, anthropometric, and biochemical data were obtained in accordance with standard procedures. Multiple diagnostic criteria were used to characterise diabetes, prediabetes, and obesity. Diagnostic concordance was evaluated using Venn diagrams and UpSet plots, and independent associated factors were identified using multivariate logistic regression.

**Results:**

The prevalence of obesity, hypertension, prediabetes, and diabetes was 45.46%, 40.19%, 29.54%, and 13.40%, respectively. Women and middle-aged adults (45–59 years) were most impacted. There was significant heterogeneity in classification criteria, with just 9.28% of obese participants and 2.36% of diabetic participants being diagnosed by all diagnostic criteria. While the absence of childhood obesity was associated with lower odds of obesity (aOR = 0.43, 95% CI: 0.30–0.61), diabetes (aOR = 0.64, 95% CI: 0.42–0.99), and hypertension (aOR = 0.63, 95% CI: 0.46–0.88), older age (≥60 years) was strongly associated with obesity (aOR = 2.54, 95% CI: 1.51–4.32) and hypertension (aOR = 4.82, 95% CI: 3.06–7.68). Low physical activity was significantly linked to hypertension (aOR = 1.73, 95% CI: 1.16–2.62), and elevated triglycerides were associated with diabetes (aOR = 2.16, 95% CI: 1.21–3.75) and hypertension (aOR = 1.84, 95% CI: 1.13–3.04).

**Conclusions:**

The burden of cardiometabolic diseases among Ghana’s informal workers is disproportionately high and diagnostically diverse. The findings emphasise the critical need for sex- and occupation-sensitive health interventions, life-course prevention strategies, and context-specific diagnostic guidelines to reduce cardiometabolic disease risk among informal workers in LMICs.

## Introduction

Cardiometabolic diseases such as obesity, hypertension, diabetes, and dyslipidaemia have reached epidemic proportions worldwide [1,2], with low- and middle-income countries (LMICs) bearing a disproportionate share of the burden [3,4]. In sub-Saharan Africa, the rising incidence of these diseases alongside the persistent burden of infectious diseases is driving a rapid epidemiological change [5], with important occupational health implications for workers in LMICs, who are largely informal and exposed to socioeconomic contexts that promote the onset and persistence of cardiometabolic diseases [6,7].

The informal economy, characterised by small, unregistered businesses and unprotected labour, is a substantial portion of the global workforce, especially in LMICs [8–10]. This sector employs more than 89% of the working population in Ghana, encompassing farmers, fisherfolk, artisans, traders, drivers, and other occupational groupings that function outside of formal regulatory frameworks [11,12]. Despite its critical role, this sector is plagued by hazardous work conditions, lack of social security, absence of, and disregard for occupational health and safety protections, and limited access to healthcare [13–15]. For these workers, achieving basic household consumption goals often takes precedence over health-related goals [16,17]. Thus, they may be disproportionately affected by non-communicable diseases (NCDs) such as cardiometabolic diseases due to the combination of financial limitations, low health literacy, and occupational hazards such as prolonged sedentary behaviour of traders, irregular eating habits, limited access to healthcare, psychosocial stress from unstable income, and physically demanding work without sufficient rest intervals [6,7,13,18]. Despite the growing burden of these cardiometabolic diseases, their exact distribution within Ghana’s informal workforce remains poorly understood.

In Ghana, previous literature has identified diabetes mellitus (6.8%), hyperuricaemia (9.3%), obesity (15.1%), hypertension (26.1%), and dyslipidaemia (34.4%) as major contributors to the cardiometabolic disease burden [19]. However, these estimates are primarily based on facility-based research or general population surveys, with little representation of informal sector workers who may show unique epidemiological patterns. The lack of targeted research on this population limits the development of workplace health interventions and contextually appropriate preventive strategies [6,13,20].

Accurate disease diagnosis is further complicated by the lack of consensus on African-tailored diagnostic criteria [21–24]. Various anthropometric measures for obesity, including body mass index (BMI), waist circumference (WC), waist-to-hip ratio (WHR), and waist-to-height ratio (WHtR), show varying sensitivity and specificity across populations [25,26], with little data on their concordance in sub-Saharan African populations [27]. Likewise, abnormal glucose tolerance can be diagnosed using fasting blood sugar (FBS), fasting plasma glucose (FPG), 2-hour postprandial glucose (2hPG), or glycated haemoglobin (HbA1c), each with distinct diagnostic thresholds [4,28]. This diagnostic heterogeneity complicates disease burden estimation and cross-study comparability [22–24,29,30].

Beyond occupational and diagnostic factors, early-life exposures play a vital role in adult cardiometabolic disease risk [31,32]. Childhood obesity has emerged as a possible determinant of adult cardiometabolic diseases, with longitudinal studies showing enduring metabolic effects that continue into adulthood [32]. However, data on the prevalence and effects of childhood obesity on adult cardiometabolic outcomes in Ghanaian populations remain inadequate.

This study addresses these knowledge gaps by comprehensively characterising the cardiometabolic profile of Ghanaian informal sector workers. Drawing on the social determinants of health framework, this study situates occupational exposures, socioeconomic status, and early-life influences as central drivers of cardiometabolic disease risk. Specifically, the study sought to ascertain the prevalence of diabetes, hypertension, obesity, and prediabetes, assess the overlap of multiple diagnostic and classification criteria, and determine the lifestyle, occupational, sociodemographic, and biochemical factors linked to cardiometabolic disease in this understudied population. The findings offer important epidemiological data to inform public health initiatives, workplace health regulations, and resource allocation strategies aimed at reducing the cardiometabolic disease burden among informal workers in LMICs.

## Materials and methods

### Study design

This cross-sectional study among Ghanaian workers in the informal sector sought to comprehensively evaluate the cardiometabolic profiles of individuals working in various informal occupations in the Cape Coast Metropolis. The study received ethical approval from the University of Cape Coast Institutional Review Board (UCCIRB/EXT/2022/27), and all participants of the study provided written informed consent. The data collection procedures adhered to the Declaration of Helsinki. Participation was voluntary and each participant signed informed consent and had the right to withdraw anytime they felt uncomfortable.

### Population

This study analysed data on Ghanaian informal workers from the larger CarMeR study. A multistage sampling technique was used to select participants from urban, peri-urban, and rural communities within the Cape Coast Metropolis. Using mapping data from the recent Ghana census [11], the study area was divided into clusters from which 13 communities were selected, and eligible participants were then recruited from each community using systematic sampling with random replacement. The sampling technique, general inclusion and exclusion criteria, as well as the justification, have been detailed in the CarMeR study protocol (https://doi.org/10.3389/fendo.2024.1337895) [33]. For this study, data on ostensibly healthy individuals with no prior diagnosis of cardiometabolic disease who were actively engaged in informal sector employment were analysed. Thus, a total of 948 informal workers distributed across five major occupation categories including drivers, farmers, fisherfolk, artisans, and traders were included in the study. This specific population was selected due to the inherent nature of their occupations making them susceptible to cardiometabolic disease risks, that can lead to diseases and even death [6,13]. Participation rate could not be ascertained, precluding characterisation of non-participation selection effects.

### Inclusion and exclusion criteria

Participants were eligible to participate if they were aged 25 years or older, actively engaged in the informal sector within the Cape Coast Metropolis, and provided written informed consent. Eligible occupational categories included traders, fisherfolk, artisans, farmers, and drivers. Exclusion criteria comprised pregnant or breastfeeding women, individuals with cognitive or psychological conditions that would interfere with their ability to complete questionnaires or take part in data collection activities, and individuals who declined to undergo the necessary anthropometric and biochemical assessments. All glycaemic and cardiometabolic assessments were conducted at the Cardiometabolic Epidemiology Research Laboratory (CERL) at the University of Cape Coast, Ghana.

### Data collection procedure

Prior to the commencement of the study, permission for community entry was sought from the relevant community leaders. The goals, advantages, and risks of the study were explained to the volunteers before the data collection was done. Data collection comprised sociodemographic, lifestyle, anthropometric, physiological, and biochemical data. The specific tests were selected for this study as they are the standard markers of cardiometabolic disease [34]. All biochemical analyses were done at the Cardiometabolic Epidemiology Research Laboratory. Data collection for this study was conducted between 23/08/2023 to 06/02/2025.

### Sociodemographic and lifestyle assessment

To ascertain the socio-demographic and lifestyle characteristics of the participants, structured questionnaires were administered by trained research assistants to collect data on lifestyle factors, childhood obesity history, and sociodemographic characteristics (age, sex, occupation, education level, and marital status). Childhood obesity was assessed retrospectively using a validated body silhouette scale from the Third Harvard Growth Study [35], where participants selected 1 of 9 somatotypes that best represented their body outline during childhood. This approach served as a proxy indicator of early-life body size. Responses were dichotomised for analysis, with participants selecting beyond the fifth silhouette classified as having childhood obesity. To aid recall, interviewers provided contextual prompts that enabled participants to think about their childhood body size. This approach has been validated for retrospective body size assessment in adult populations and reduces reliance on verbal interpretation of weight status terminology [35,36]. Additionally, sensitivity analyses excluding childhood obesity yielded consistent results (S2 – S4 Tables). Furthermore, physical activity levels of the participants were assessed with the International Physical Activity Questionnaire (IPAQ), and categorised as high, moderate, and low [37].

### Anthropometric measurements

All anthropometric measurements were taken repeatedly by qualified personnel following established protocols, and the average value was used for final analysis. Body weight was measured with a calibrated digital scale to the nearest 0.1 kg, with participants wearing light clothing and no shoes. Using a stadiometer, height was measured to the closest 0.1 cm. Weight in kilogrammes divided by height in metres squared (kg/m^2^) was used to compute the body mass index (BMI), the most widely used measure of overall adiposity [38,39].

Using a non-stretchable measuring tape, the waist circumference (WC) was determined at the midpoint between the iliac crest and the lower costal margin as an index of central adiposity and visceral fat accumulation. Hip circumference was measured at the maximum circumference over the buttocks. To determine the waist-to-hip ratio (WHR), WC was divided by hip circumference. Also, waist-to-height ratio (WHtR), a height-adjusted central adiposity measure that has demonstrated screening utility across diverse populations [40], was determined by dividing WC by height [38].

Based on the criteria of the World Health Organisation (WHO), individuals were classified as obese if they met any of the criteria of having a BMI ≥ 30 kg/m^2^, WC ≥ 94 cm in men and ≥ 80 cm in women, WHR of  ≥ 0.90 in men and ≥ 0.85 in women, or a WHtR of  > 0.50 [38]. All four indices were included to enable a comprehensive evaluation of diagnostic concordance across measures capturing distinct dimensions of adiposity.

### Blood pressure measurement

Blood pressure was measured using a validated automated sphygmomanometer (Sinocare BSX 516 Blood Pressure Monitor, Sinocare Inc, China), after participants rested in a seated position for at least five minutes. Measurements on the right arm were taken three times at two-minute intervals, and the average of the last two readings was used for the analysis [33]. Systolic blood pressure ≥ 140 mmHg, diastolic blood pressure ≥ 90 mmHg, or the use of antihypertensive medication were considered indicators of hypertension [41].

### Biochemical assessments

Venous blood samples were collected after an overnight fast of at least 8 hours for measurement of high-density lipoprotein (HDL) cholesterol, triglycerides, glycated haemoglobin (HbA1c), and fasting plasma glucose (FPG). Fasting blood sugar (FBS) was measured at the point of care from capillary blood using an ACCU-CHECK INSTANT glucometer device (Roche, Germany, 2019). Both FBS and FPG were included to assess the agreement between point-of-care and laboratory methods for glucose measurement. Additionally, participants underwent an oral glucose tolerance test (OGTT) using 82.5 g of dextrose monohydrate (equivalent to 75 g of anhydrous glucose) with blood samples collected two hours after the glucose load to determine 2-hour postprandial glucose (2hPG) levels [33]. HDL cholesterol, triglycerides, FPG, and 2hPG were analysed with the Mindray BS-240 Blood Chemistry Analyser (Mindray, China, August 2022) and HbA1c with the Wondfo Finecare III Plus Fluorescent Immunoassay analyser (Wondfo, China, June 2022).

In this study, prediabetes was classified as FBS between 5.6 and 6.9 mmol/L (100–125 mg/dL), FPG between 5.6 and 6.9 mmol/L (100–125 mg/dL), HbA1c between 5.7% and 6.4%, or 2hPG between 7.8 and 11.0 mmol/L (140–199 mg/dL). Diabetes was classified as FBS ≥ 7.0 mmol/L (126 mg/dL), FPG ≥ 7.0 mmol/L (126 mg/dL), 2hPG ≥ 11.1 mmol/L (200 mg/dL), or HbA1c ≥ 6.5%. Additionally, HDL cholesterol levels of < 1.0 mmol/L (40 mg/dL) in men and < 1.3 mmol/L (50 mg/dL) in women were classified as low, and triglyceride levels of ≥ 1.7 mmol/L (150 mg/dL) were characterised as elevated [42,43].

### Statistical analyses

Data were analysed using the R software (version 4.5.1). Descriptive statistics were presented as frequencies and percentages. Venn diagrams and UpSet plots (UpSetR package) were used to visualise the overlap among different diagnostic criteria for diabetes, prediabetes, and obesity [44]. For each condition, participants were classified as positive if they met any one of the respective diagnostic criteria, in order to maximise case identification and to enable the concordance analyses that constituted a primary objective of this study. Additionally, pairwise inter-criterion agreement analyses were conducted for each pair of classification criteria. One criterion in each pair was designated as the comparator solely as an analytical reference. Agreement was summarised using positive percent agreement, negative percent agreement, positive predictive value (PPV), negative predictive value (NPV), overall percent agreement, and a concordance statistic (area under the receiver operating characteristic curve, AUC), all with 95% confidence intervals. Multivariate logistic regression analysis was used to assess the independent associated factors of the study outcomes (obesity, prediabetes, diabetes, and hypertension). Variables including age, sex, occupation, marital status, educational level, childhood obesity, physical activity level, HDL cholesterol status, and triglyceride status were assessed in a univariable analysis. Variables found to be significant or considered clinically important based on the literature were included in the multivariable models. All models were adjusted simultaneously for the retained covariates to account for potential confounding. Interaction terms did not reach statistical significance and were not retained in the final models. The results were presented as adjusted odds ratios (aOR) with 95% confidence intervals (CI). The regression models included all participants regardless of weight status, with individuals without the outcome of interest serving as the reference group for each analysis. Statistical significance was set at p < 0.05 for all analyses in the study.

### Ethics approval

This study was approved by the University of Cape Coast Institutional Review Board with the reference number UCCIRB/EXT/2022/27. All participants gave written informed consent before commencement of the study.

## Results

### Prevalence of cardiometabolic diseases

Among the 948 participants included in the analysis, the prevalence of cardiometabolic diseases was substantial. Obesity was the most common disease, affecting 431 individuals (45.46%), followed by hypertension affecting 381 participants (40.19%). A total of 280 participants (29.54%) were diagnosed with prediabetes, while 127 participants (13.40%) were diagnosed with diabetes.

### Sociodemographic, lifestyle, and biochemical characteristics

Middle-aged adults (45–59 years) constituted the majority across all diseases, representing 52.23% − 55.91% of affected individuals. A pronounced sex disparity was observed, with females comprising 88.40% of participants with obesity, 78.74% with diabetes, 70.60% with hypertension, and 64.64% with prediabetes.

Traders were the most represented occupational category across all diseases (47.14% − 62.65%) followed by fisherfolk for prediabetes (22.14%) and hypertension (22.05%), and artisans for diabetes (21.26%) and prediabetes (20.00%). Low physical activity was the predominant pattern among participants across all four diseases (54.29% − 62.20%). While the majority of participants did not report childhood obesity (65.89% − 73.57%), a notable proportion (26.43% − 34.11%) had a history of childhood obesity.

Regarding lipid profiles, low HDL cholesterol was observed in approximately half of those with diabetes (51.97%) and obesity (54.76%), and in 48.82% and 42.50% of those with hypertension and prediabetes respectively. Fewer participants had elevated triglycerides, ranging from 7.86% with prediabetes to 15.75% with diabetes.

The sociodemographic, lifestyle, and biochemical characteristics of participants with each cardiometabolic disease are further shown in Table 1.

**Table 1:** Sociodemographic, lifestyle, and biochemical characteristics of participants with obesity, prediabetes, diabetes, and hypertension.

Characteristics	Obesity (n = 431)	Prediabetes (n = 280)	Diabetes (n = 127)	Hypertension (n = 381)
**Age Category**				
Young Adults (25–44 years)	74 (17.17%)	56 (20.0%)	20 (15.75%)	45 (11.81%)
Middle-aged adults (45–59 years)	234 (54.29%)	148 (52.86%)	71 (55.91%)	199 (52.23%)
Old-aged adults (60 years +)	123 (28.54%)	76 (27.14%)	36 (28.35%)	137 (35.96%)
**Sex**				
Female	381 (88.40%)	181 (64.64%)	100 (78.74%)	269 (70.60%)
Male	50 (11.60%)	99 (35.36%)	27 (21.26%)	112 (29.40%)
**Occupation**				
Artisan	56 (12.99%)	56 (20.00%)	27 (21.26%)	70 (18.37%)
Driver	3 (0.70%)	6 (2.14%)	1 (0.79%)	6 (1.57%)
Farmer	41 (9.51%)	24 (8.57%)	14 (11.02%)	37 (9.71%)
Fisherfolk	61 (14.15%)	62 (22.14%)	18 (14.17%)	84 (22.05%)
Trader	270 (62.65%)	132 (47.14%)	67 (52.76%)	184 (48.29%)
**Childhood Obesity**				
Yes	147 (34.11%)	74 (26.43%)	42 (33.07%)	110 (28.87%)
No	284 (65.89%)	206 (73.57%)	85 (66.93%)	271 (71.13%)
**Physical Activity Levels**				
High	56 (12.99%)	54 (19.29%)	19 (14.96%)	50 (13.12%)
Low	254 (58.93%)	152 (54.29%)	79 (62.20%)	229 (60.10%)
Moderate	121 (28.07%)	74 (26.43%)	29 (22.83%)	102 (26.77%)
**HDL cholesterol**				
Adequate HDL	195 (45.24%)	161 (57.50%)	61 (48.03%)	195 (51.18%)
Low HDL	236 (54.76%)	119 (42.50%)	66 (51.97%)	186 (48.82%)
**Triglycerides**				
Normal	382 (88.63%)	258 (92.14%)	107 (84.25%)	337 (88.45%)
Elevated	49 (11.37%)	22 (7.86%)	20 (15.75%)	44 (11.55%)

Notes: Percentages may not total 100 due to rounding; HDL – High-density lipoprotein.

### Diagnostic criteria concordance

Substantial heterogeneity was observed in the classification of participants depending on the diagnostic criteria used for obesity (BMI, WC, WHR, WHtR), prediabetes (FBS, FPG, 2hPG, HbA1c), and diabetes (FBS, FPG, 2hPG, HbA1c).

### Obesity classification

Among the 431 participants classified as obese by at least one criterion, BMI identified 231 persons, WHR identified 246, WHtR identified 249, and WC identified only 86 individuals as a stand-alone criterion (Fig 1). Only 40 participants (9.28%) met all four anthropometric criteria for obesity. The most prevalent two-criterion overlap was BMI and WHtR (n = 167), whereas BMI combined with WHR and WHtR identified 70 people.

**Fig 1:**
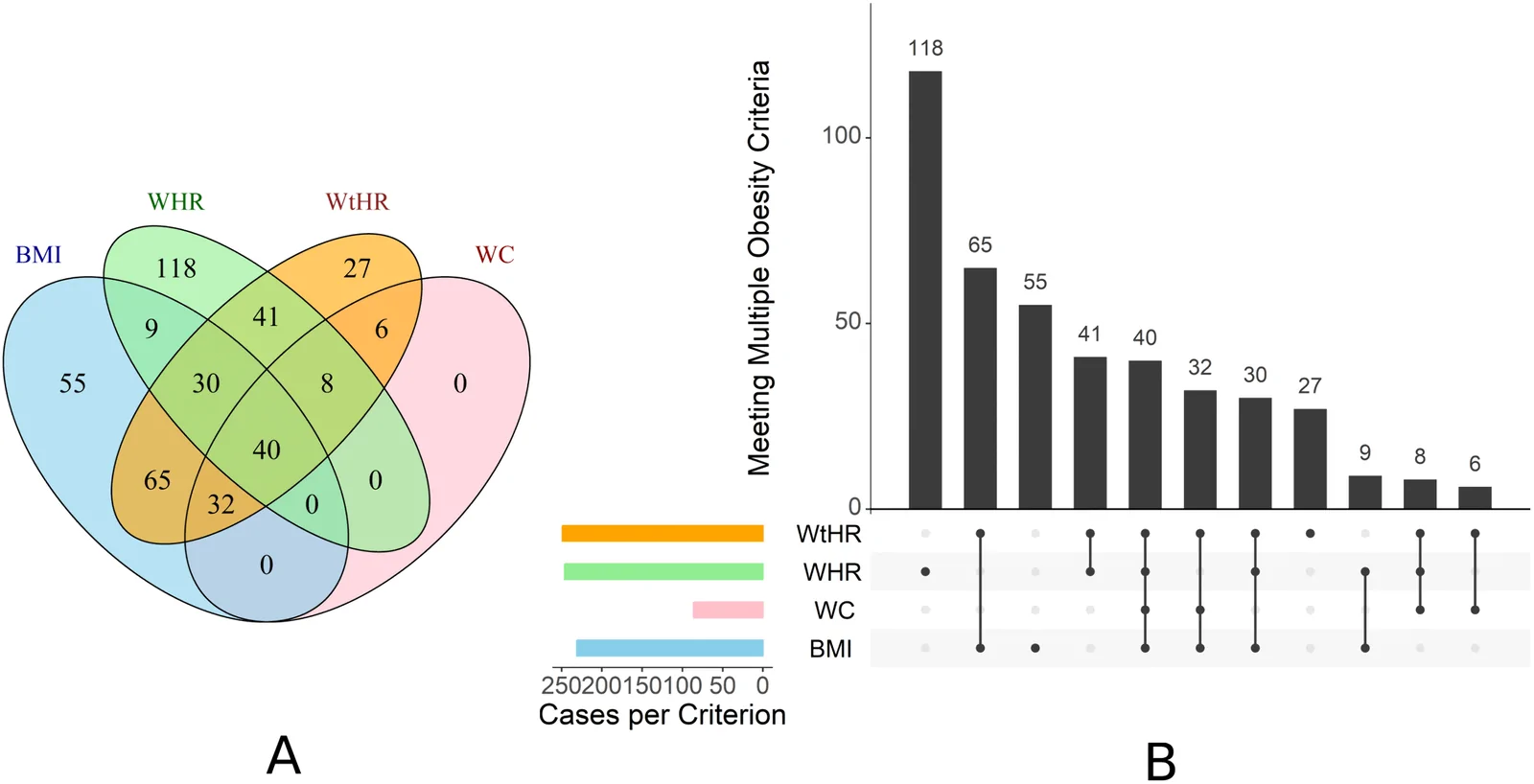
Venn diagram (A) and UpSet plot (B) demonstrating the overlap of classification criteria for obesity [body mass index (BMI), waist circumference (WC), waist-to-hip ratio (WHR), and waist-to-height ratio (WHtR)]. The figures represent the number of individuals classified as obese by each criterion and their intersections.

### Prediabetes diagnosis

Of the 280 participants diagnosed with prediabetes, FBS identified the greatest number of cases (n = 150), followed by FPG (n = 138), HbA1c (n = 118), and 2hPG (n = 71) as stand-alone criteria (Fig 2). Notably, only one participant was diagnosed by all four tests, indicating minimal overlap between the various diagnostic techniques. The most common two-test overlap was FBS and FPG (n = 79). There was minimal concordance among three-test combinations, with the highest being 19 participants diagnosed by FBS, FPG, and 2hPG.

**Fig 2:**
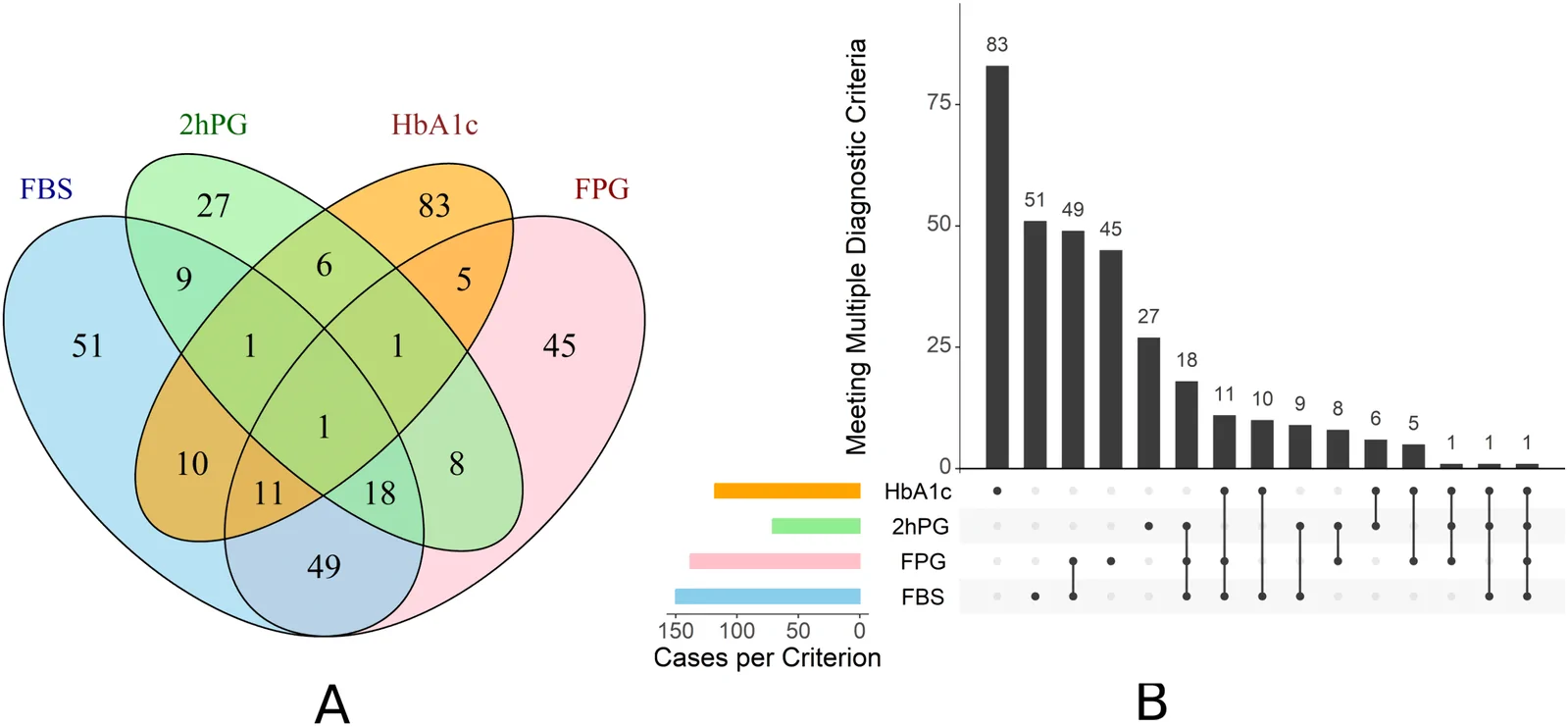
Venn diagram (A) and UpSet plot (B) demonstrating the overlap of prediabetes diagnostic tests [fasting blood sugar (FBS), fasting plasma glucose (FPG), 2-hour postprandial glucose (2hPG), and glycated haemoglobin (HbA1c)]. The figures represent the number of individuals diagnosed as prediabetic by each test and their intersections.

### Diabetes diagnosis

Among the 127 participants with diabetes, HbA1c identified the most cases as a stand-alone criterion (n = 87), followed by 2hPG (n = 38), FPG (n = 36), and FBS (n = 32) (Fig 3). Only three individuals (2.36%) were identified by all four diagnostic tests. There was concordance between the two test combinations of FBS and FPG (n = 26), FPG and 2hPG (n = 25), and FBS and 2hPG (n = 21). HbA1c demonstrated the least concordance with other criteria in two- and three-test combinations.

**Fig 3:**
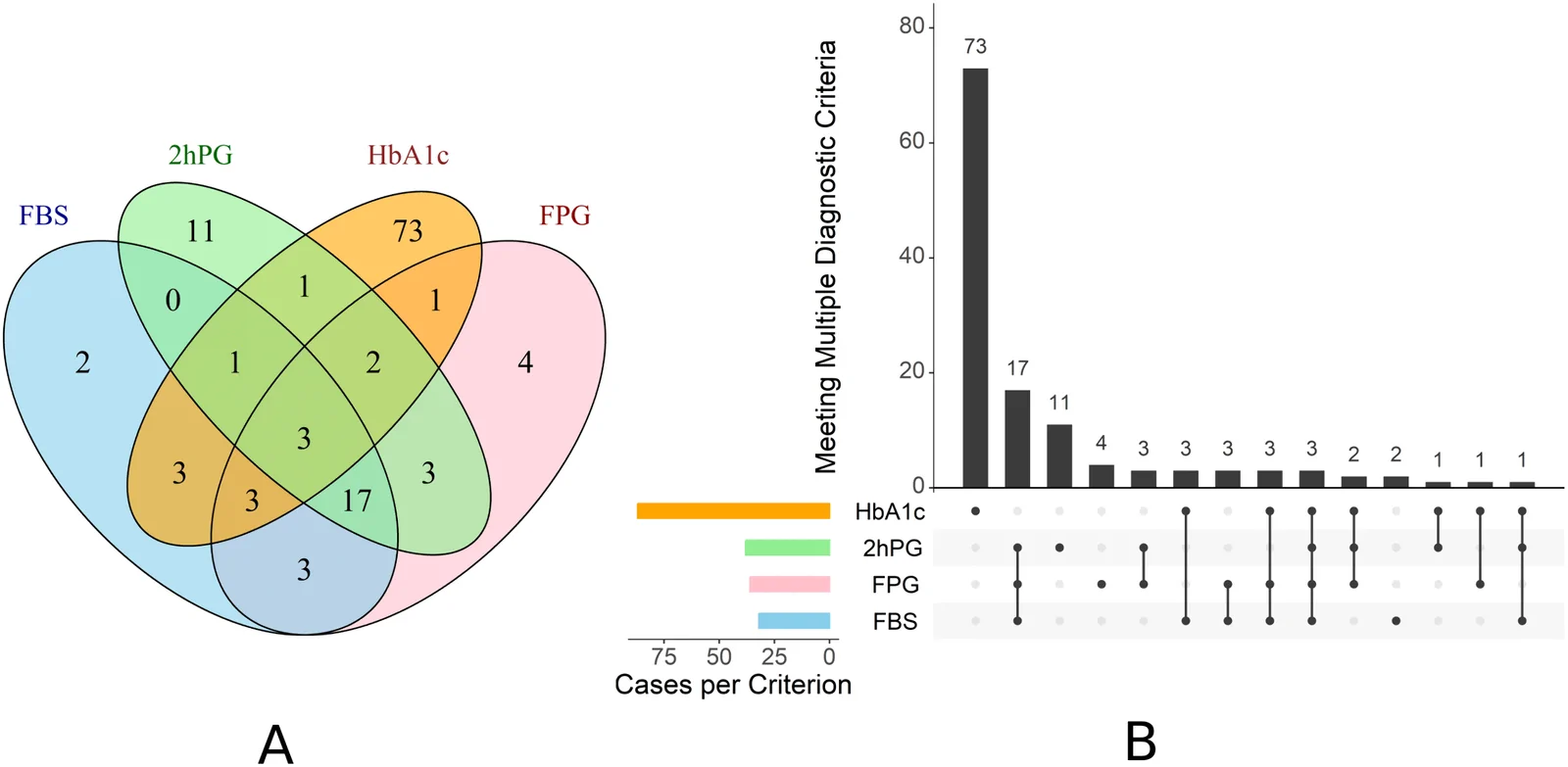
Venn diagram (A) and UpSet plot (B) demonstrating the overlap of diabetes diagnostic tests [fasting blood sugar (FBS), fasting plasma glucose (FPG), 2-hour postprandial glucose (2hPG), and glycated haemoglobin (HbA1c)]. The figures represent the number of individuals diagnosed as diabetic by each test and their intersections.

### Inter-criterion agreement analyses

Pairwise agreement analyses showed variable concordance among classification criteria (Fig 4).

**Fig 4:**
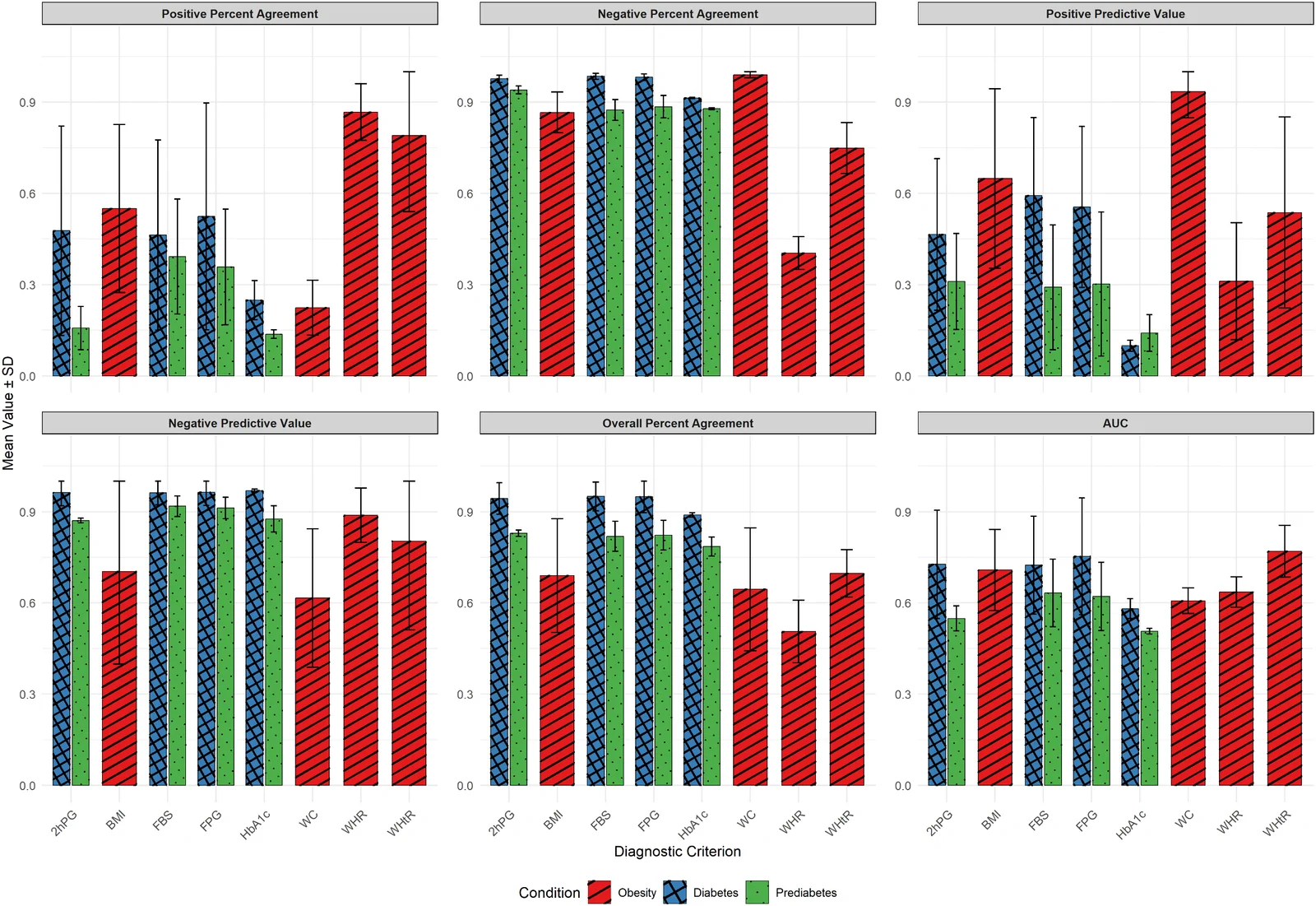
Inter-criterion agreement for obesity, prediabetes, and diabetes classification, summarised as positive percent agreement, negative percent agreement, positive predictive value (PPV), negative predictive value (NPV), overall percent agreement, and a concordance statistic (AUC). The figure presents bar charts representing mean values ± standard deviation for each diagnostic criterion, stratified by disease (diabetes, prediabetes, obesity).

For obesity, WC and BMI demonstrated strong agreement (AUC = 0.826, 95% CI: 0.785–0.868), with BMI classifying 83.7% of WC-defined obese individuals as obese (positive percent agreement). WHtR identified all WC-defined obese individuals (100% positive agreement) and aligned closely with BMI (AUC = 0.804, 95% CI: 0.777–0.832). In contrast, WHR demonstrated weaker agreement with other criteria (AUC = 0.562–0.673; negative percent agreement of 37–47% relative to BMI or WC), indicating it identifies a partly different subgroup rather than performing less accurately.

For prediabetes, glucometer-based FBS and laboratory FPG showed the strongest agreement (AUC = 0.726–0.742), classifying a moderate proportion of each other’s cases concordantly (positive percent agreement 52.7–57.2%) while agreeing closely on non-cases (negative percent agreement 91.2–92.6%). In contrast, HbA1c showed poor agreement with all other criteria (AUC = 0.501–0.521) with positive percent agreement as low as 7.6–19.5%.

Regarding diabetes, FBS and FPG demonstrated excellent concordance (AUC = 0.858–0.901), with glucometer-based FBS as the analytical reference. FPG concordantly classified 81.2% of cases (positive percent agreement) and 98.9% of non-cases (negative percent agreement). 2hPG also showed good agreement with fasting glucose measures (AUC = 0.823–0.840). However, HbA1c showed poor concordance with all fasting and post-load glucose tests (AUC = 0.522–0.614), with positive percent agreement of only 8.0–31.2%. The inter-criterion agreement analyses are comprehensively illustrated in Fig 4.

### Factors associated with cardiometabolic diseases

Multivariate logistic regression was conducted to identify factors independently associated with each cardiometabolic outcome (Table 2). Model diagnostics indicated no significant multicollinearity using variance inflation factor (VIF), and adequate calibration across all models after a Hosmer-Lemeshow goodness-of-fit test. While the prediabetes model showed limited differentiation (AUC = 0.565), the obesity model demonstrated good discrimination (AUC = 0.797), followed by hypertension (AUC = 0.680) and diabetes (AUC = 0.646). Full diagnostics are presented in S1 Table.

**Table 2:** Sociodemographic, lifestyle, and clinical factors associated with obesity, prediabetes, diabetes and hypertension.

Characteristics	Obesity (n = 431)		Prediabetes (n = 280)		Diabetes (n = 127)		Hypertension (n = 381)	
	aOR (95% CI)	*p*	aOR (95% CI)	*p*	aOR (95% CI)	*p*	aOR (95% CI)	*p*
**Age Category**								
25-44 years (Ref)	1.00	–	1.00	–	1.00	–	1.00	–
45–59 years	1.61 (1.07 - 2.42)	0.022	0.98 (0.67 - 1.43)	0.900	1.55 (0.90 - 2.76)	0.124	2.19 (1.48 - 3.27)	<0.001
60 years +	2.54 (1.51 - 4.32)	<0.001	1.10 (0.71 - 1.72)	0.675	1.70 (0.90 - 3.29)	0.107	4.82 (3.06 - 7.68)	<0.001
**Sex**								
Female (Ref)	1.00	–	1.00	–	1.00	–	1.00	–
Male	0.13 (0.08 - 0.20)	<0.001	1.15 (0.79 - 1.69)	0.458	0.56 (0.32 - 0.96)	0.037	0.76 (0.53 - 1.11)	0.154
**Occupation**								
Artisan (Ref)	1.00	–	1.00	–	1.00	–	1.00	–
Driver	1.47 (0.55 - 3.79)	0.434	1.20 (0.41 - 3.15)	0.724	0.94 (0.29 - 2.84)	0.554	1.11 (0.37 - 3.00)	0.839
Farmer	3.09 (1.42 - 7.25)	0.007	1.43 (0.76 - 2.65)	0.260	1.13 (0.50 - 2.48)	0.763	0.95 (0.52 - 1.73)	0.876
Fisherfolk	1.63 (1.02 - 2.61)	0.042	1.68 (1.07 - 2.64)	0.024	0.65 (0.33 - 1.26)	0.207	1.30 (0.84 - 2.01)	0.245
Trader	1.55 (0.96 - 2.48)	0.070	1.48 (0.97 - 2.30)	0.072	0.81 (0.46 - 1.45)	0.472	1.02 (0.67 - 1.54)	0.940
**Childhood Obesity**								
Yes (Ref)	1.00	–	1.00	–	1.00	–	1.00	–
No	0.43 (0.30 - 0.61)	<0.001	0.88 (0.63 - 1.23)	0.447	0.64 (0.42 - 0.99)	0.041	0.63 (0.46 - 0.88)	0.006
**Physical Activity Levels**								
High (Ref)	1.00	–	1.00	–	1.00	–	1.00	–
Low	1.24 (0.78 - 1.95)	0.357	0.88 (0.59 - 1.31)	0.526	1.30 (0.75 - 2.35)	0.371	1.73 (1.16 - 2.62)	0.008
Moderate	1.47 (0.90 - 2.41)	0.125	0.87 (0.57 - 1.34)	0.517	0.95 (0.51 - 1.80)	0.866	1.41 (0.91 - 2.19)	0.126
**HDL cholesterol**								
Adequate HDL (Ref)	1.00	–	1.00	–	1.00	–	1.00	–
Low HDL	0.94 (0.66 - 1.34)	0.732	0.83 (0.62 - 1.11)	0.206	1.13 (0.76 - 1.67)	0.555	1.05 (0.79 - 1.39)	0.740
**Triglycerides**								
Normal (Ref)	1.00	–	1.00	–	1.00	–	1.00	–
Elevated	1.76 (0.89 - 3.74)	0.123	0.91 (0.53 - 1.51)	0.714	2.16 (1.21 - 3.75)	0.007	1.84 (1.13 - 3.04)	0.016

Note: aOR – Adjusted Odds Ratio; CI – Confidence Intervals; HDL – High-density lipoprotein. Reference categories (Ref): Age (25–44 years); Sex (Female); Occupation (Artisan); Childhood obesity (Yes); Physical activity (High); HDL cholesterol (Adequate); Triglycerides (Normal).

### Age-related associations

Advancing age was significantly associated with both obesity and hypertension (Table 2). Compared with younger adults, both middle-aged (aOR = 1.61, 95% CI: 1.07–2.42, p = 0.022) and older adults (aOR = 2.54, 95% CI: 1.51–4.32, p < 0.001) had higher odds of obesity. A similar age pattern was observed for hypertension, with middle-aged adults (aOR = 2.19, 95% CI: 1.48–3.27, p < 0.001) and older adults (aOR = 4.82, 95% CI: 3.06–7.68, p < 0.001) showing elevated odds. Age was not significantly associated with prediabetes or diabetes.

### Sex-specific associations

The male sex was independently associated with significantly lower odds of obesity (aOR = 0.13, 95% CI: 0.08–0.20, p < 0.001) and diabetes (aOR = 0.56, 95% CI: 0.32–0.96, p = 0.037) compared with females. There were no significant associations between sex and either hypertension or prediabetes (Table 2).

### Occupational associated factors

Differential associations between occupational groups and cardiometabolic diseases were identified. Compared with artisans, farmers (aOR = 3.09, 95% CI: 1.42–7.25, p = 0.007) and fisherfolk (aOR = 1.63, 95% CI: 1.02–2.61, p = 0.042) had significantly higher odds of obesity, while traders showed a similar but non-significant trend (aOR = 1.55, 95% CI: 0.96–2.48, p = 0.070). Fisherfolk also had elevated odds of prediabetes (aOR = 1.68, 95% CI: 1.07–2.64, p = 0.024). Occupation was not significantly associated with diabetes or hypertension (Table 2).

### Childhood obesity

Absence of childhood obesity was significantly associated with lower odds of adult obesity (aOR = 0.43, 95% CI: 0.30–0.61, p < 0.001), diabetes (aOR = 0.64, 95% CI: 0.42–0.99, p = 0.041), and hypertension (aOR = 0.63, 95% CI: 0.46–0.88, p = 0.006). There was a similar trend with prediabetes, although this was not statistically significant (Table 2).

### Physical activity associations

Hypertension was significantly associated with low levels of physical activity (aOR = 1.73, 95% CI: 1.16–2.62, p = 0.008). Physical activity was not significantly associated with obesity, diabetes, or prediabetes in the adjusted models (Table 2).

### Associations with lipid measures

HDL cholesterol and triglyceride levels were not significantly associated with obesity in the adjusted models. Elevated triglycerides were, however, significantly associated with diabetes (aOR = 2.16, 95% CI: 1.21–3.75, p = 0.007) and hypertension (aOR = 1.84, 95% CI: 1.13–3.04, p = 0.016) (Table 2).

## Discussion

This study provides a comprehensive characterisation of the cardiometabolic health profile of Ghanaian informal sector workers, a significant but understudied population. The prevalences of diabetes (13.40%), prediabetes (29.54%), obesity (45.46%), and hypertension (40.19%) among informal workers are significantly higher than Ghana’s general national averages [4,19], highlighting the silent epidemic developing in this sector. These rates are consistent with the precarious nature of informal work, characterised by psychosocial stress, limited access to healthcare, and occupational hazards promoting unhealthy dietary practices and sedentary lifestyles [6,13].

The disproportionate burden of obesity and diabetes among women warrants careful interpretation. The sex-obesity association (aOR = 0.13) likely reflects multiple converging factors. First, methodological effects, as the WHO anthropometric criteria employ substantially lower WC and WHR thresholds for women, potentially inflating classification rates among women given the criterion used. Second, residual occupational confounding may be present, as the trader category, which is predominantly female and accounted for the majority of the obesity cases in this sample, combines with broad occupational coding that cannot capture within-occupation variation in sedentary, food-proximate work. Consequently, sex and occupational exposure are substantially entangled rather than independently estimated. Third, gendered sociocultural constraints, as women in this setting often face reduced autonomy and may prioritise family health over their own [45,46]. The disparity therefore reflects a convergence of these factors [47], rather than any single cause. The magnitude of this association may therefore be overstated by residual confounding and by differential classification arising from sex-specific anthropometric thresholds, and the aOR of 0.13 should not be read as a precise estimate of the sex difference in adiposity.

Only 9.28% of obese participants satisfied all four anthropometric criteria and just 2.36% of participants with diabetes were diagnosed by all four glucose tests, consistent with previous concordance studies [22,30,48]. The pairwise comparisons reflect diagnostic agreement rather than true accuracy, as no widely recognised gold standard exists for obesity or dysglycaemia diagnosis in African populations. Each criterion captures a different physiological construct, where BMI represents overall adiposity, WC and WHtR indicate central adiposity, and FBS/FPG and HbA1c represent fasting glucose homeostasis and average glycaemic exposure respectively. This means discordance reflects identification of partially overlapping high-burden subpopulations rather than criterion failure. For obesity, BMI and WHtR showed close alignment with WC, whereas WHR showed poor agreement [22,30,48]. For dysglycaemia, glucometer-based FBS and laboratory FPG showed the strongest concordance for both prediabetes and diabetes [49], indicating that point-of-care glucometers reproduced laboratory-based fasting classification with reasonable consistency and are therefore potentially feasible for field screening, with additional support from the high concordance between 2hPG and fasting measures. The poor alignment of HbA1c with the fasting and post-load glucose criteria is among the most relevant findings of this study, and is unlikely to reflect classification heterogeneity alone. Several factors are known to decouple HbA1c from prevailing glycaemia in sub-Saharan African populations. These include structural haemoglobin variants such as HbS and HbC traits, which shorten erythrocyte survival or interfere with the assay [50], and anaemia, which alters HbA1c independently of glucose [51]. Iron deficiency, particularly common among women of reproductive age who predominated in this cohort, raises HbA1c at any given glucose level and may partly explain why HbA1c flagged cases the glucose-based tests did not [52]. Also, ancestry-related differences in glycation biology, where individuals of African descent can show higher HbA1c for a given mean glucose, mean that thresholds derived elsewhere may not transfer directly [53], while incomplete standardisation of point-of-care assays and their susceptibility to variant haemoglobins add further measurement variance [50]. As haemoglobin phenotype, iron and inflammatory status, as well as pre-analytical handling and cross-platform assay agreement were not assessed here, their contributions cannot be quantified and the net direction of bias remains uncertain. Nonetheless, these mechanisms offer a biologically coherent account of the discordance and support cautious interpretation of HbA1c as a standalone screening criterion in this population, while lending plausibility to the potential utility of fasting glucose-based point-of-care approaches for field screening in LMIC settings, pending population-specific and assay-specific validation [54]. These patterns imply that overreliance on a single diagnostic tool may result in significant under-or over-diagnosis [30,55–57].

Based on the concordance patterns, combining BMI with WC or WHtR for obesity screening, and using point-of-care glucometers for fasting glucose-based dysglycaemia screening, appear comparable enough to merit evaluation in field settings, while HbA1c should be interpreted cautiously as a standalone screening criterion in this population until population-specific and assay-specific validation is available. These represent observations on the comparability of alternative classification criteria rather than definitive clinical protocols, as prospective validation against hard clinical endpoints remains necessary. Thus, the urgent need for validated Africa-specific cut-off points to ensure precise burden estimation and cross-study comparability cannot be overstated [58,59].

Advancing age was strongly associated with obesity and hypertension, consistent with cumulative lifetime exposures [60,61]. The predominance of middle-aged adults across all diseases is concerning, as this economically productive segment’s disease burden affects household economic stability and intergenerational poverty cycles [62–64]. Occupational patterns were evident, with farmers and fisherfolk showing significantly higher odds of obesity compared with artisans, and fisherfolk also showing higher odds of prediabetes, possibly reflecting seasonal food insecurity-abundance cycles among farmers [65], and irregular, high-calorie diets combined with physically demanding but metabolically inconsistent work among fisherfolk [66]. Occupation was not significantly associated with diabetes or hypertension, suggesting that these may be more strongly driven by age or lifestyle factors, or that occupational categories were too broad to capture specific exposure patterns. This resonates with evidence emphasising that cardiometabolic disease risk exposure is shaped by a constellation of factors, including unhealthy diets, sedentary routines, and psychosocial stress, that compound the threat for those in irregular, unprotected work [7]. The working conditions of the informal sector likely limit opportunities for health-promoting physical activity [6], as reflected in the notable relationship between low physical activity and hypertension [67,68]. Elevated triglycerides were significantly associated with diabetes and hypertension consistent with established cardiometabolic disease pathways [69,70], though associations with obesity did not reach significance after adjustment. HDL cholesterol was not independently associated with any outcome.

The findings are consistent with the life-course epidemiology model in population health [71]. Participants reporting no childhood obesity showed substantially lower odds of adult obesity, diabetes, and hypertension, consistent with long-lasting metabolic consequences of early-life exposures [31,32]. However, the somatotype recall method provides a proxy rather than an objective measure, and recall bias may vary systematically by age and sex, with older participants facing longer recall periods, and sociocultural norms around body size perception possibly influencing somatotype selection, particularly where larger childhood body size is viewed positively. Combined with the inability to exclude shared familial, genetic, or environmental determinants, these associations should be regarded as hypothesis-generating rather than as evidence of causal life-course effects, and are intended to motivate, not pre-empt, prospective investigation. Childhood obesity was retained in the models primarily to adjust for potential life-course confounding rather than as a primary exposure of comparable standing to objectively measured variables. The sensitivity analyses excluding this variable (S2–S4 Tables) confirmed that all other associations remained stable, demonstrating that the main findings are not contingent on this proxy measure. A cautious, hypothesis-generating interpretation is further supported by the fact that these analyses do not correct for measurement error within the childhood-obesity variable itself. As a result, residual recall bias and differential memory accuracy across age groups (older participants recalling over longer intervals, and body-size perception shaped by sociocultural norms) may remain substantial. If these relationships represent true life-course effects, they would have implications for early-life interventions aimed at reducing the burden of adult cardiometabolic diseases in LMICs [72], though addressing the knowledge gap regarding childhood obesity prevalence and determinants in African populations remains a prerequisite.

### Public health and policy implications

These findings have significant implications for occupational health policy, resource allocation, and healthcare delivery in LMICs. The disproportionate burden of cardiometabolic disease among informal workers, who frequently lack social protection and employer-sponsored insurance, demonstrates a significant equity issue [9,14]. With the majority of Ghana’s workforce in the informal sector [11], addressing these structural determinants requires multi-level interventions [6,13]. The diagnostic concordance findings suggest that practical, low-cost screening using BMI with a measuring tape and point-of-care glucometers may warrant evaluation in this setting, as such tools are deployable at market centres, lorry parks, and landing sites through trained community health workers within Ghana’s existing CHPS framework. Whether such an approach improves case detection or downstream cardiometabolic outcomes would require prospective evaluation. Expanded coverage under the National Health Insurance Scheme should also be prioritised.

The diagnostic heterogeneity has implications for prevalence estimation, clinical management, and health system planning. Clinically, discordant classifications create diagnostic uncertainty, such as when an individual is classified as obese by WHR but not by BMI, or as having diabetes by HbA1c but not fasting glucose. At the health system level, programmes employing different criteria may identify different individuals for follow-up, potentially leading to missed cases or inefficient resource use. However, these observations require confirmation through population-specific validation studies [21,73].

At the policy level, informal sector health priorities should be integrated into NCD action plans through revised health financing, workplace health regulations adapted to informal employment, and partnerships with trade associations for health promotion delivery [7]. The sex disparities emphasise the need for sex-responsive approaches that account for sex variations in exposures, time constraints, and obstacles to accessing healthcare. For female traders, who bore the highest burden, screening days organised through market women’s associations could offer a feasible delivery mechanism. The associations between childhood obesity and adult outcomes highlight the significance of early prevention through school-based nutrition programmes [74], improved food environments [75], and physical activity promotion [76], leveraging existing community structures [77].

The convergence of high disease burden, identifiable high-burden subgroups, and feasible low-cost screening tools creates a concrete opportunity for action. Integrating cardiometabolic screening into routine occupational health outreach and ensuring equitable insurance coverage would represent practical steps toward reducing the cardiometabolic burden among the most vulnerable yet economically vital populations in Ghana and sub-Saharan Africa.

### Limitations

Despite its insightful findings, the study was not without limitations. The cross-sectional nature of the study precludes causal inference. While analysers were calibrated according to manufacturers’ specifications and internal quality control procedures were followed, formal external quality assurance was not available. Enrolling only ostensibly healthy workers with no prior diagnosis likely excluded severe or treated disease and may have introduced a healthy-worker survivor effect, rendering the reported prevalences conservative estimates of previously undetected disease rather than total community burden. The inter-criterion agreement analyses reflect agreement between alternative classification criteria rather than true diagnostic accuracy since no single reference standard exists, and any derived screening recommendations require prospective validation against clinical outcomes. Self-reported childhood obesity remains a proxy measure subject to recall bias that may differ systematically across age groups, and the associations it yielded should be considered hypothesis-generating. Prospective studies with objectively measured early-life exposures are needed to clarify these associations. The metropolitan study setting limits generalisability to informal workers elsewhere in Ghana and sub-Saharan Africa. Additionally, residual confounding from genetic and environmental exposures cannot be excluded. BMI-stratified analyses and studies incorporating objective physical activity measurements and genetic data could provide a more comprehensive understanding of cardiometabolic disease aetiology in this population.

## Conclusions

This study reveals that informal sector workers in Ghana carry a disproportionate and poorly characterised burden of cardiometabolic disease. The health of this economically critical group is compromised by diagnostic inconsistency arising from conflicting criteria, as well as the strong associations between age, lifestyle, early-life factors, occupation, and cardiometabolic outcomes. These findings highlight the alarming prevalence of cardiometabolic diseases and identify the factors associated with cardiometabolic disease in this demographic. The significant diagnostic heterogeneity across the different classification criteria highlights important methodological challenges for accurate disease surveillance and clinical management in African populations, and necessitates immediate efforts to reach a consensus on population-appropriate diagnostic guidelines. These observations offer crucial evidence for developing tailored public health initiatives, guiding the allocation of resources, and influencing workplace health policies in order to mitigate the alarming rise in cardiometabolic disease in LMICs. Future efforts should focus on the standardisation of diagnostic approaches and the implementation of multi-level strategies that address risks throughout the life course, from childhood to the unique context of the informal workplace. Prospective longitudinal studies are needed to establish causal relationships and inform evidence-based interventions for this vulnerable population.

## Supporting information

S1 Table Model Diagnostic Summary for Multivariate Logistic Regression. (DOCX)

S2 Table Odds Ratio Comparison Between Analyses with and without Childhood Obesity. (DOCX)

S3 Table Model Discrimination and Multicollinearity Analysis for the Exclusion of Childhood Obesity. (DOCX)

S4 Table Summary Statistical Inference for Analyses Performed. (DOCX)
