# Supplementary material for: Prevalence and risk factors of cardiometabolic disease among informal sector workers in Cape Coast, Ghana: Insights from the CarMeR study
Source: PLoS One. 2026 Sep 29;21(9):e0359530. doi: 10.1371/journal.pone.0359530 (PMC13623084; doi:10.1371/journal.pone.0359530)
Supplement: Supplementary file 1 [file pone.0359530.s001.docx]

**Model Performance Measures for Multivariate Logistic Regression Analysis**

**Table 1: Model Diagnostic Summary for Multivariate Logistic Regression**

| **Outcome** | **H-L χ²** | **H-L p-value** | **AUC (95% CI)** | **Max VIF** |
| --- | --- | --- | --- | --- |
| Obesity | 6.218 | 0.623 | 0.797 (0.764-0.830) | 1.756 |
| Prediabetes | 14.937 | 0.060 | 0.565 (0.526-0.604) | 1.655 |
| Diabetes | 3.672 | 0.885 | 0.646 (0.594-0.698) | 1.468 |
| Hypertension | 7.823 | 0.451 | 0.680 (0.646-0.715) | 1.625 |

Note: H-L χ² - Hosmer-Lemeshow Goodness-of-Fit; AUC - area under the receiver operating characteristic curve; VIF – Variance Inflation Factor. H-L p-value > 0.05 indicates adequate model calibration; AUC ≥ 0.70 indicates acceptable discrimination; VIF < 5 indicates no significant multicollinearity.
