# Supplementary material for: Prevalence and risk factors of cardiometabolic disease among informal sector workers in Cape Coast, Ghana: Insights from the CarMeR study
Source: PLoS One. 2026 Sep 29;21(9):e0359530. doi: 10.1371/journal.pone.0359530 (PMC13623084; doi:10.1371/journal.pone.0359530)
Supplement: Supplementary file 2 [file pone.0359530.s002.docx]

**Sensitivity analysis excluding childhood obesity as a factor (odds ratio stability)**

We conducted a sensitivity analysis to assess whether the inclusion of childhood obesity as a covariate influenced our findings. All multivariable logistic regression models were refit after excluding the childhood obesity variable. The results for odds ratio stability are displayed below:

**Table 2: Odds Ratio Comparison Between Analyses with and without Childhood Obesity**

| **Outcome** | **Predictor** | **OR (95% CI) With childhood obesity** | **OR (95% CI) Without childhood obesity** | **% Change** | **p-value (With)** | **p-value (Without)** | **CI Overlap** | **Direction** |
| --- | --- | --- | --- | --- | --- | --- | --- | --- |
| Obesity | Age: 45–59 years | 1.61 (1.07-2.42) | 1.58 (1.05-2.38) | -1.30% | 0.271 | 0.312 | Yes | Same |
| Obesity | Age: 60 years + | 2.54 (1.51-4.32) | 2.47 (1.47-4.19) | -2.80% | 0.018 | 0.020 | Yes | Same |
| Obesity | Sex: Male | 0.13 (0.08-0.20) | 0.12 (0.08-0.19) | -7.80% | 0 | 0 | Yes | Same |
| Obesity | Profession: Driver | 1.47 (0.55-3.79) | 1.34 (0.50-3.44) | -8.70% | 0.568 | 0.547 | Yes | Same |
| Obesity | Profession: Farmer | 3.09 (1.42-7.25) | 3.04 (1.39-7.09) | -1.90% | 0.015 | 0.012 | Yes | Same |
| Obesity | Profession: Fisherfolk | 1.63 (1.02-2.61) | 1.60 (1.00-2.56) | -1.50% | 0.294 | 0.310 | Yes | Same |
| Obesity | Profession: Trader | 1.55 (0.96-2.48) | 1.48 (0.92-2.36) | -4.40% | 0.002 | 0.03 | Yes | Same |
| Obesity | Physical activity: Low | 1.24 (0.78-1.95) | 1.08 (0.69-1.68) | -12.60% | 0.105 | 0.428 | Yes | Same |
| Obesity | Physical activity: Moderate | 1.47 (0.90-2.41) | 1.42 (0.87-2.32) | -3.10% | 0.026 | 0.034 | Yes | Same |
| Obesity | Low HDL cholesterol | 0.94 (0.66-1.34) | 0.97 (0.69-1.38) | 3.10% | 0.05 | 0.108 | Yes | Same |
| Obesity | Elevated triglycerides | 1.76 (0.89-3.74) | 1.84 (0.94-3.89) | 4.80% | 0.028 | 0.003 | Yes | Same |
| Prediabetes | Age: 45–59 years | 0.98 (0.67-1.43) | 0.97 (0.67-1.42) | -0.20% | 0.9 | 0.893 | Yes | Same |
| Prediabetes | Age: 60 years + | 1.10 (0.71-1.72) | 1.09 (0.70-1.71) | -0.80% | 0.675 | 0.699 | Yes | Same |
| Prediabetes | Sex: Male | 1.15 (0.79-1.69) | 1.13 (0.78-1.64) | -2.20% | 0.458 | 0.526 | Yes | Same |
| Prediabetes | Profession: Driver | 1.20 (0.41-3.15) | 1.17 (0.40-3.07) | -2.20% | 0.724 | 0.756 | Yes | Same |
| Prediabetes | Profession: Farmer | 1.43 (0.76-2.65) | 1.41 (0.75-2.62) | -1.10% | 0.26 | 0.274 | Yes | Same |
| Prediabetes | Profession: Fisherfolk | 1.68 (1.07-2.64) | 1.68 (1.07-2.64) | 0.10% | 0.024 | 0.024 | Yes | Same |
| Prediabetes | Profession: Trader | 1.48 (0.97-2.30) | 1.47 (0.96-2.28) | -0.70% | 0.072 | 0.077 | Yes | Same |
| Prediabetes | Physical activity: Low | 0.88 (0.59-1.31) | 0.85 (0.58-1.26) | -3.10% | 0.526 | 0.419 | Yes | Same |
| Prediabetes | Physical activity: Moderate | 0.87 (0.56-1.34) | 0.86 (0.56-1.33) | -0.70% | 0.517 | 0.495 | Yes | Same |
| Prediabetes | Low HDL cholesterol | 0.83 (0.62-1.11) | 0.83 (0.62-1.12) | 0.80% | 0.206 | 0.224 | Yes | Same |
| Prediabetes | Elevated triglycerides | 0.91 (0.53-1.51) | 0.92 (0.54-1.53) | 1.30% | 0.714 | 0.751 | Yes | Same |
| Diabetes | Age: 45–59 years | 1.55 (0.90-2.76) | 1.52 (0.89-2.70) | -1.90% | 0.124 | 0.138 | Yes | Same |
| Diabetes | Age: 60 years + | 1.70 (0.90-3.29) | 1.63 (0.87-3.15) | -4.00% | 0.107 | 0.134 | Yes | Same |
| Diabetes | Sex: Male | 0.56 (0.32-0.96) | 0.52 (0.30-0.89) | -6.70% | 0.037 | 0.018 | Yes | Same |
| Diabetes | Profession: Driver | 0.53 (0.03-2.84) | 0.50 (0.03-2.62) | -7.30% | 0.554 | 0.506 | Yes | Same |
| Diabetes | Profession: Farmer | 1.13 (0.50-2.48) | 1.08 (0.48-2.37) | -4.00% | 0.763 | 0.841 | Yes | Same |
| Diabetes | Profession: Fisherfolk | 0.65 (0.33-1.26) | 0.67 (0.34-1.29) | 3.20% | 0.207 | 0.238 | Yes | Same |
| Diabetes | Profession: Trader | 0.81 (0.46-1.45) | 0.81 (0.46-1.43) | -0.50% | 0.472 | 0.458 | Yes | Same |
| Diabetes | Physical activity: Low | 1.30 (0.75-2.35) | 1.15 (0.67-2.05) | -11.50% | 0.371 | 0.625 | Yes | Same |
| Diabetes | Physical activity: Moderate | 0.95 (0.51-1.80) | 0.92 (0.49-1.75) | -2.90% | 0.866 | 0.795 | Yes | Same |
| Diabetes | Low HDL cholesterol | 1.13 (0.76-1.67) | 1.16 (0.78-1.71) | 2.50% | 0.555 | 0.472 | Yes | Same |
| Diabetes | Elevated triglycerides | 2.16 (1.21-3.75) | 2.25 (1.26-3.89) | 4.00% | 0.007 | 0.005 | Yes | Same |
| Hypertension | Age: 2 | 2.19 (1.48-3.27) | 2.15 (1.47-3.21) | -1.50% | 0 | 0 | Yes | Same |
| Hypertension | Age: 3 | 4.82 (3.06-7.68) | 4.62 (2.95-7.35) | -4.00% | 0 | 0 | Yes | Same |
| Hypertension | Sex: 2 | 0.76 (0.53-1.11) | 0.71 (0.49-1.02) | -6.90% | 0.154 | 0.068 | Yes | Same |
| Hypertension | Profession: 2 | 1.11 (0.37-3.00) | 1.04 (0.35-2.77) | -6.90% | 0.839 | 0.947 | Yes | Same |
| Hypertension | Profession: 3 | 0.95 (0.52-1.73) | 0.93 (0.51-1.68) | -2.90% | 0.876 | 0.799 | Yes | Same |
| Hypertension | Profession: 4 | 1.30 (0.84-2.01) | 1.31 (0.85-2.02) | 1.00% | 0.245 | 0.225 | Yes | Same |
| Hypertension | Profession: 8 | 1.02 (0.67-1.54) | 1.00 (0.66-1.51) | -1.60% | 0.94 | 0.998 | Yes | Changed |
| Hypertension | Physical activity: Low | 1.73 (1.16-2.62) | 1.54 (1.04-2.30) | -11.10% | 0.008 | 0.032 | Yes | Same |
| Hypertension | Physical activity: Moderate | 1.41 (0.91-2.19) | 1.36 (0.89-2.11) | -3.10% | 0.126 | 0.161 | Yes | Same |
| Hypertension | Low HDL cholesterol | 1.05 (0.79-1.39) | 1.08 (0.81-1.43) | 2.60% | 0.74 | 0.609 | Yes | Same |
| Hypertension | High triglycerides | 1.84 (1.13-3.04) | 1.92 (1.18-3.16) | 4.10% | 0.016 | 0.009 | Yes | Same |

Note: Of the 44 predictor-outcome comparisons, 93.2% changed by less than 10% after removing childhood obesity. No predictor crossed the null value (OR=1.0) in either direction, and 97.7% maintained the same direction of association.
