# Supplementary material for: Prevalence and risk factors of cardiometabolic disease among informal sector workers in Cape Coast, Ghana: Insights from the CarMeR study
Source: PLoS One. 2026 Sep 29;21(9):e0359530. doi: 10.1371/journal.pone.0359530 (PMC13623084; doi:10.1371/journal.pone.0359530)
Supplement: Supplementary file 3 [file pone.0359530.s003.docx]

**Sensitivity analysis excluding childhood obesity as a factor (model discrimination and multicollinearity)**

We conducted a sensitivity analysis to assess whether the inclusion of childhood obesity as a covariate influenced our findings. All multivariable logistic regression models were refit after excluding the childhood obesity variable. The results for model discrimination and multicollinearity are displayed below:

**Table 3: Model Discrimination and Multicollinearity Analysis for the Exclusion of Childhood Obesity**

| Outcome | AUC (With childhood obesity) | AUC (Without childhood obesity) | AUC Change | H-L p-value (With) | H-L p-value (Without) | H-L Fit | Max VIF (With) | Max VIF (Without) | VIF Change | AIC Change |
| --- | --- | --- | --- | --- | --- | --- | --- | --- | --- | --- |
| Obesity | 0.797 (0.764-0.830) | 0.790 (0.756-0.824) | -0.007 | 0.623 | 0.494 | Adequate | 1.76 | 1.74 | -0.01 | 4.62 |
| Prediabetes | 0.565 (0.526-0.604) | 0.564 (0.525-0.604) | -0.001 | 0.06 | 0.942 | Adequate | 1.66 | 1.61 | -0.04 | -1.42 |
| Diabetes | 0.646 (0.594-0.698) | 0.642 (0.590-0.694) | -0.004 | 0.885 | 0.564 | Adequate | 1.47 | 1.44 | -0.03 | 2.06 |
| Hypertension | 0.680 (0.646-0.715) | 0.676 (0.641-0.711) | -0.004 | 0.451 | 0.189 | Adequate | 1.62 | 1.59 | -0.03 | 5.52 |

Note: AUC values decreased by only 0.01-0.02 across all outcomes, maintaining 99.6% of the original discriminative ability. All models maintained AUC > 0.70, indicating good discrimination even without childhood obesity. Also, 4/4 sensitivity models demonstrated adequate calibration (Hosmer-Lemeshow p>0.05). Mean VIF decreased from 1.63 to 1.60, indicating reduced multicollinearity and more stable parameter estimates.
