# Supplementary material for: Prevalence and risk factors of cardiometabolic disease among informal sector workers in Cape Coast, Ghana: Insights from the CarMeR study
Source: PLoS One. 2026 Sep 29;21(9):e0359530. doi: 10.1371/journal.pone.0359530 (PMC13623084; doi:10.1371/journal.pone.0359530)
Supplement: Supplementary file 4 [file pone.0359530.s004.docx]

**Sensitivity analysis excluding childhood obesity as a factor (statistical inference)**

We conducted a sensitivity analysis to assess whether the inclusion of childhood obesity as a covariate influenced our findings. All multivariable logistic regression models were refit after excluding the childhood obesity variable. The results for statistical inference are displayed below:

**Table 4: Summary Statistical Inference for Analyses Performed**

| **Robustness Indicator** | **Value** | **Interpretation** |
| --- | --- | --- |
| AUC maintained (>90% of original) | 99.60% | Excellent discrimination maintained |
| Models with adequate fit (HL p>0.05) | 4/4 (100%) | Adequate calibration in all models |
| ORs with <10% change | 93.20% | Minimal confounding effect |
| ORs with <20% change | 100% | No meaningful confounding |
| ORs with overlapping 95% CI | 100% | Statistically equivalent estimates |
| Statistical significance unchanged | 100% | Inference unchanged |
| Direction of association unchanged | 97.70% | No sign flipping |
| Mean VIF (with obesity) | 1.63 | Moderate collinearity |
| Mean VIF (without obesity) | 1.6 | Low collinearity |
| VIF improvement | 0.03 | Reduced multicollinearity |
| AIC improvement (mean) | 2.7 | More parsimonious models |

Note: 100.0% of predictors maintained the same statistical significance (p<0.05 threshold), and confidence intervals overlapped in 100.0% of comparisons, indicating statistically equivalent estimates.

These findings demonstrate that our results are robust and not contingent upon the inclusion of childhood obesity in the multivariable models. Childhood obesity acts as a minimal confounder, and its exclusion does not substantively alter the associations between the remaining covariates and the outcomes of interest.
